# Behavioural and Neural Responses to Facial Disfigurement

**DOI:** 10.1038/s41598-019-44408-8

**Published:** 2019-05-29

**Authors:** Franziska Hartung, Anja Jamrozik, Miriam E. Rosen, Geoffrey Aguirre, David B. Sarwer, Anjan Chatterjee

**Affiliations:** 1 Center for Cognitive Neuroscience Department of Neurology at the School of Medicine, University of Pennsylvania Goddard Laboratory 3710, Hamilton Walk, 19104 Philadelphia, PA USA; 2 Penn Center for Neuroaesthetics Department of Neurology at the School of Medicine, University of Pennsylvania Goddard Laboratory 3710, Hamilton Walk, 19104 Philadelphia, PA USA; 3 Center for Obesity Research and Education College of Public Health, Department of Social and Behavioral Sciences, Temple University 1301 Cecil B. Moore Avenue, 19122 Philadelphia, PA USA

**Keywords:** Perception, Human behaviour

## Abstract

Faces are among the most salient and relevant visual and social stimuli that humans encounter. Attractive faces are associated with positive character traits and social skills and automatically evoke larger neural responses than faces of average attractiveness in ventral occipito-temporal cortical areas. Little is known about the behavioral and neural responses to disfigured faces. In two experiments, we tested the hypotheses that people harbor a disfigured is bad bias and that ventral visual neural responses, known to be amplified to attractive faces, represent an attentional effect to facial salience rather than to their rewarding properties. In our behavioral study (N = 79), we confirmed the existence of an implicit ‘*disfigured is bad*’ bias. In our functional MRI experiment (N = 31), neural responses to photographs of disfigured faces before treatment evoked greater neural responses within ventral occipito-temporal cortex and diminished responses within anterior cingulate cortex. The occipito-temporal activity supports the hypothesis that these areas are sensitive to attentional, rather than reward properties of faces. The relative deactivation in anterior cingulate cortex, informed by our behavioral study, may reflect suppressed empathy and social cognition and indicate evidence of a possible neural mechanism underlying dehumanization.

## Introduction

Physical appearance has a profound impact on a person’s life. Beautiful people are preferred and enjoy many advantages compared to average-looking people^1^. While conceptually orthogonal, the correlation of attractiveness and positive character traits indicates the prevalence of a ‘beautiful is good’ stereotype^2–4^. This stereotype might be innate^5^. Attractive people are seen as more trustworthy, socially competent, dominant, better adjusted, more capable in school and work, and also receive greater rewards and lesser punishments than their average looking peers^5–7^. Adults and children ascribe desirable personality traits to attractive faces of adults and children and discriminate against unattractive faces even if they are friends and family members^5^. Attractiveness and trustworthiness judgments are consistent across cultures^5^ and are made extremely quickly^3,8^. Longer exposure to a face does not attenuate these biases and instead only consolidates people’s confidence in a judgement already made^3^. Attractiveness also highly influences visual exploration of faces^9,10^.

In this study we examine a corollary to the ‘beautiful is good’ stereotype, that an automatic ‘disfigured is bad’ stereotype also exists. People with facial disfigurement are stigmatized and are often targets of discrimination. Looking at disfigured faces makes observers feel less happy, less in control, less dominant, and more aroused^6^. People with facial disfigurements are not only perceived as less attractive and less likely to be selected as romantic partners, they are also thought of as having unfavourable personality traits (e.g., lack of emotional stability, conscientiousness), internal attributes (e.g., unhappiness, lower intelligence), social qualities (e.g., untrustworthiness, unpopularity)^6,7,11,12^ and are treated poorly in social interactions^6,11–23^. In popular culture, facial disfigurement is often used to distinguish good and evil characters^24^. Well known examples of disfigured villains are Scar in the *Lion King* (large facial scar over left eye), Freddy Krueger in *Nightmare on Elm Street* (3rd degree burns and exposed tissue), the *James Bond* villains Le Chiffre (facial scar over left eye), Emilio Largo (missing eye), Ernst Stavro Blofeld (large scar over right eye covering most of his right side of the face), and Alec Trevelyan (facial burn scars), Elle Driver in *Kill Bill* (missing eye), Two Face in the *Batman* Universe (acid scars covering the left side of his head), Hopper in *A Bug’s Life* (scar covering right eye), and the Duchess from *Alice in a Wonderland* (Macrocephaly). This ‘disfigurement is bad’ stereotype is only partially explained by lower attractiveness of disfigured faces^6^.

Attractiveness of faces –and therefore attribution of a’beauty is good’ stereotype- is highly correlated with typicality or statistical averageness of faces^25,26^. In addition to being statistical averages of groups, attractive faces are also symmetric^25^. Both facial symmetry and averageness are considered markers of physical health and influence peoples’ choices of partners^27–29^. Disfigured faces are neither typical nor average, and are usually not symmetric. They often deviate substantially from the norm. If proximity to the norm predicts positive social attributions, being ‘different’ could lead to negative evaluations. Disfigured faces might be linked to unfavourable personality traits, internal attributes, and social qualities because they are less typical and deviate from the population average. The association of disfigurement with negative attributes probably drives stigmatization and discrimination of disfigured people in social, academic, and professional contexts^6,11–23^. The stigmatization and discrimination of disfigured people likely contributes to low self-esteem^12^ and long term mental health concerns similar to other stigmatized groups that are subject to dehumanization^30^. Dehumanization deprives a person or a group of people of positive human qualities and has been shown for several stigmatized groups such as homeless people and drug addicts^31–33^. Dehumanization is used as a propaganda tool in political conflicts^34,35^. The strongest predictors of dehumanization are hypothesized to be perceived competence and warmth^31,33^. Faces rated lowest on both competence and warmth most robustly evoke dehumanization - including feelings of disgust and lack of empathy^31,33^.

Neuroimaging studies show that seeing attractive faces evokes brain responses in reward, emotion, and visual areas compared to seeing faces of average attractiveness^36–46^. Attractive faces produce activations in areas associated with reward, like the nucleus accumbens^36–38^, and orbitofrontal cortex^36,39–46^. Moreover, attractiveness correlates with increased activations in areas associated with emotion, empathy, and social cognition like the anterior cingulate cortex and medio-prefrontal cortex^37,43,47^ the latter being particularly active in tasks in which people are not making explicit attractiveness judgements^40^. Different regions of the prefrontal cortex are selectively responsive to either attractive or unattractive faces^37^ which is consistent with findings that ventral medio-prefrontal cortex processes stimulus value attributes in coordination with higher order visual areas like fusiform gyri and semantic processing areas (posterior superior temporal sulcus)^48^. Orbital frontal^46^ and medial prefrontal cortices^49^ seem to process both aesthetic and moral values and may represent the biological link between these two kinds of evaluation^49^.

Left and right amygdala seem to be sensitive to both attractive^40^ and unattractive faces^50,51^. These non-linear effects for extremes at either end of the attractiveness spectrum suggest that amygdala activation reflects sensitivity to valence intensity rather than positive or negative valence per se^26,38^. In line with the valence processing hypothesis for the functional role of amygdala, increased activation in the amygdala (bilaterally) is linked to untrustworthiness of faces^52–54^. A meta-analysis of brain activations to attractiveness and trustworthiness suggests that activation of amygdala and adjacent nucleus accumbens is driven by extremes and atypicality^26^. There is some tentative evidence that face typicality can also account for the activations in medio-prefrontal and anterior cingulate cortex^26^. The authors note that the brain networks activated in response to extremes of attractiveness and trustworthiness are remarkably similar to brain networks that process positive and negative emotions^26^.

In addition to increased brain activations in reward and emotion areas, attractive faces also evoke larger neural responses in selective visual processing areas within ventral occipito-temporal cortex (such as the fusiform face area) as compared to faces of average attractiveness^26,39,40,42,43,47,50,55,56^. These areas remain sensitive to facial attractiveness even when subjects are engaged in tasks in which attractiveness judgements are not queried explicitly. These observations have previously been interpreted as evidence that these areas also process rewards. While a reward response is one possible explanation for this amplified neural response to attractive faces, it is also possible that this reflects sensitivity to the saliency of attractive faces^39^. If this alternate hypothesis is true, other salient features, such as disfigurement, should lead to similarly amplified neural responses in visual processing areas.

Viewing faces of stigmatized groups fails to activate brain regions associated with empathy and social cognition^31,33^. Krendl and colleagues reported increased activation in anterior insula and amygdala which correlated with self-reported disgust in response to viewing faces of stigmatized groups^57^. The lack of activation in empathy and social cognition regions of the brain is postulated to be a neural correlate of dehumanization^31,33,57,58^.

Appearance clearly affects how people are viewed and treated by others. The same mechanisms that benefit attractive people in social interaction, put unattractive people at an unfair disadvantage. The effects of discriminating against people with facial disfigurement seem to extend beyond the specific effects of lower overall attractiveness and may tie in more with the pattern of results that have been shown with stigmatized groups.

The goal of the present study was to test the behavioural and brain responses to facial disfigurement and investigate whether surgical treatment mitigates these responses. In two experiments, we used a set of photograph pairs of patients with different types of facial disfigurements before and after surgical treatment of the disfigurement. In experiment one we tested if people harbour implicit biases against disfigured faces and if such implicit biases were different from consciously aware self-reported explicit biases. In a follow up functional MRI (fMRI) study, we tested differential automatic brain responses to the same picture pairs when naïve participants were engaged in an unrelated cover task. We hypothesized that people have negative biases against faces with disfigurement. For the neural responses to facial disfigurement we tested competing hypotheses: visual cortices respond to rewards per se, or visual cortices respond to salience. In addition, we expected disfigured faces to show selective responses in emotion and valence areas such as anterior insulae and amygdalae and anterior cingulate and lateral or medial prefrontal areas in line with the research reviewed above.

## Results and Discussion

**Figure 1:**
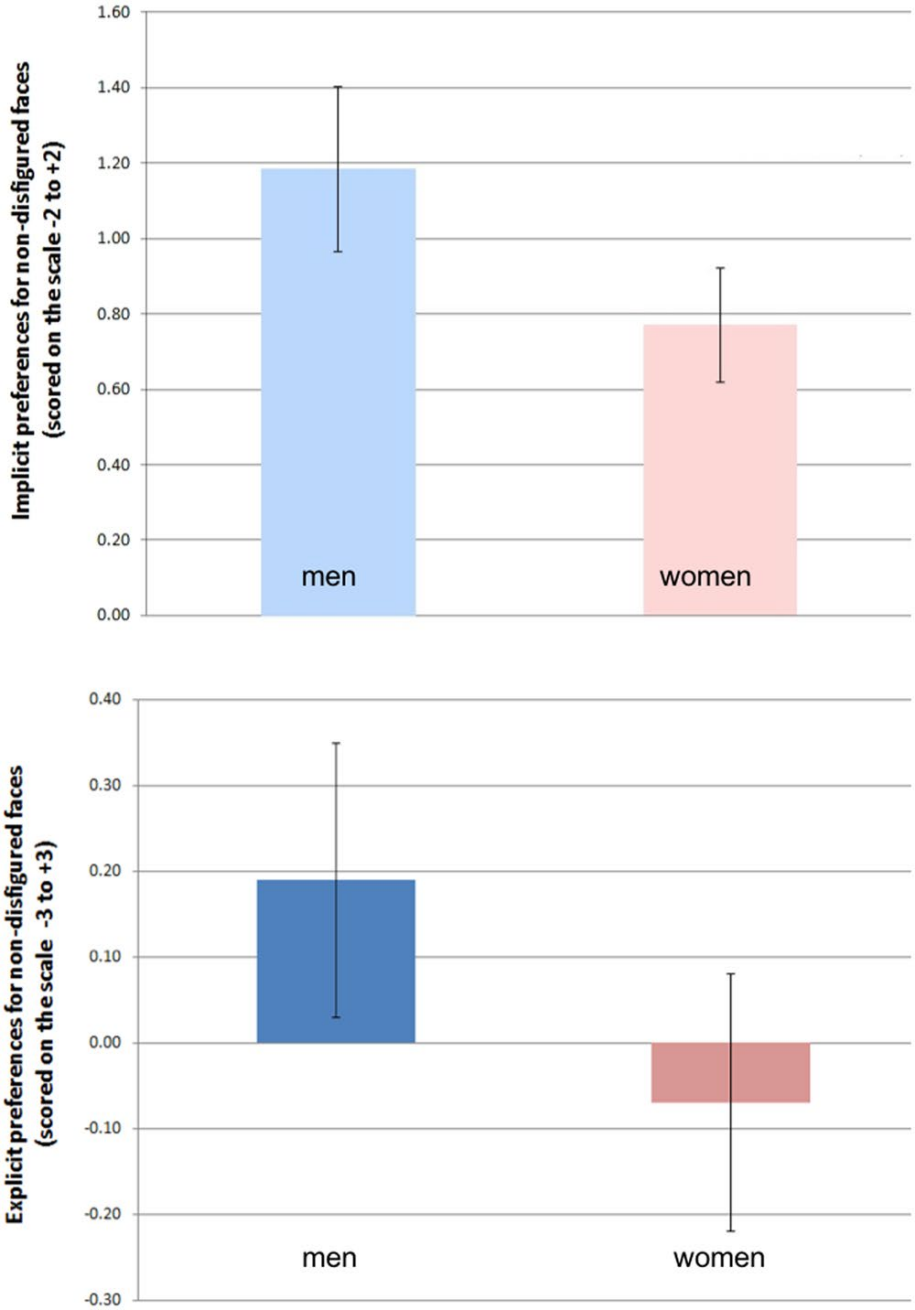
Female respondents demonstrate significantly less, although still strong, implicit preference for non-disfigured faces than male respondents. Male respondents show a moderate explicit preference for non-disfigured faces while women show no explicit preference. Error bars indicate 95% confidence intervals.

The behavioural experiment (N = 79, see method section for details) consisted of an implicit association test^59,60^ (IAT) and an explicit bias questionnaire (EBQ) to test the hypothesis that people have a negative bias for disfigured faces. For the IAT, we used a stimulus set of photographs of real patients taken before and after treatment for disfigurement. The EBQ consisted of 11 questions which query conscious biases against people with facial disfigurements (see https://osf.io/ca2u9/ for all items and data). We found no indication of an explicit bias. However, we did find that non-disfigured faces were preferred in the IAT (see Fig. 1). This bias was particularly robust for men, consistent with previous findings^5^. Prior exposure to disfigured faces did not modulate the implicit bias of individuals.

We used the same set of photographs of people before and after surgical treatment that we used in the IAT in the fMRI study (N = 31). Participants viewed these photographs and engaged in a gender judgement task. We measured neural responses to facial disfigurement to test competing hypotheses of reward versus salience in visual areas like fusiform face area. If these visual areas respond to rewards, then non-disfigured faces compared to disfigured faces would show increased activity in visual areas linked to face processing. If these visual areas respond to salience, then we should find the opposite results; disfigured faces compared to non-disfigured faces should show increased activity in these areas. Because people with facial disfigurement are likely treated as an outgroup^6^, neural patterns in response to disfigurement should be similar to previous findings investigating other stigmatized groups^31,33,57,58^. We predicted decreased activation in areas linked to social cognition such as medio-prefrontal cortex and anterior cingulate cortex, as well as increased activations in areas linked to disgust and negative emotion like anterior insula and amygdala.

**Figure 2:**
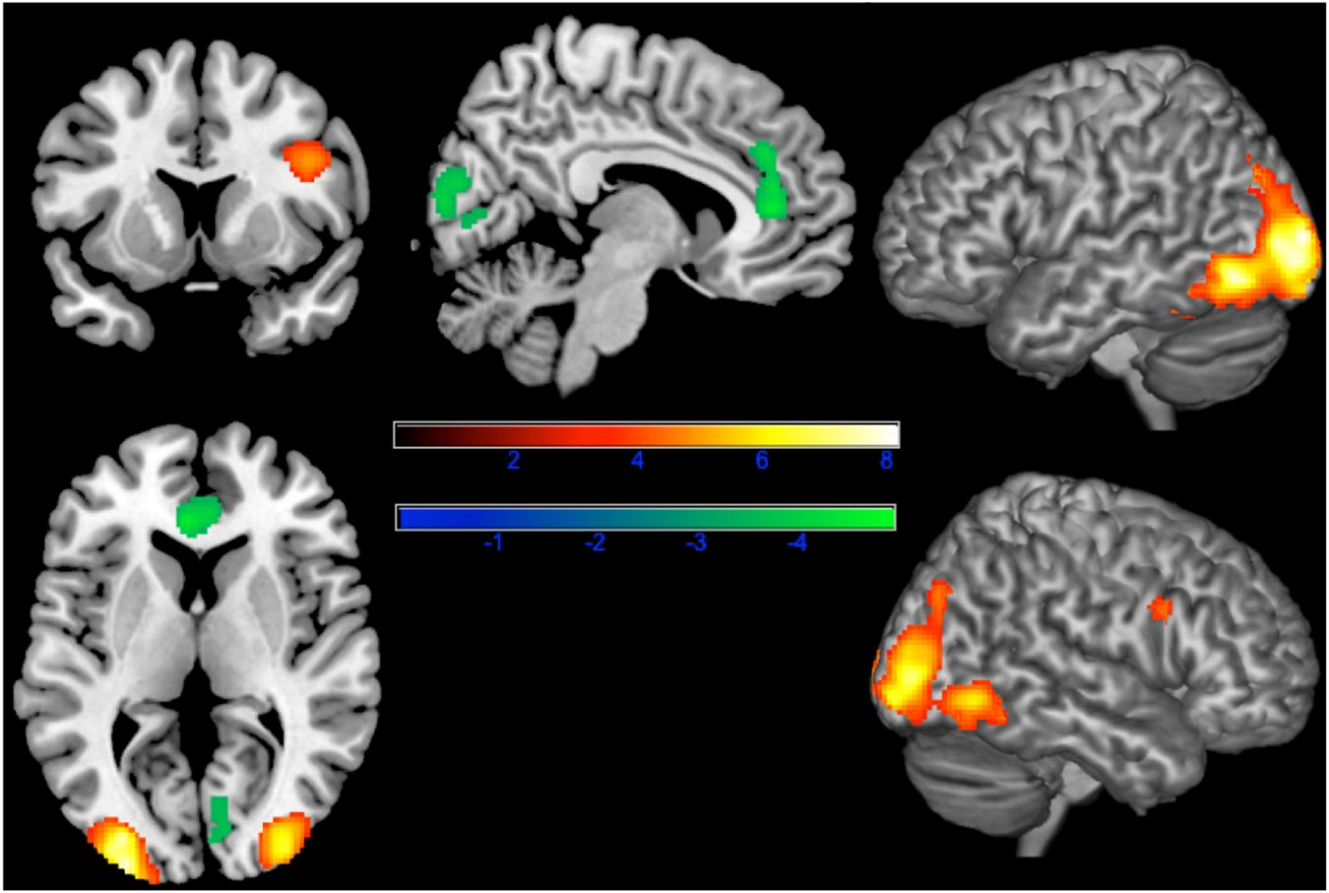
Increased activations (red yellow) and deactivations (blue-green) in response to faces before treatment. Results were corrected for multiple comparisons by familywise error correction at p < 0.05 with Monte Carlo permutation testing in SnPM with a combined cluster-voxel threshold (cluster defining threshold p < 0.001, T > 3.3852).

We found that images of people with facial disfigurement, as compared to images of the same faces after surgical treatment, evoked greater neural responses within ventral occipito-temporal cortex, particularly bilateral fusiform gyri (see Fig. 2), and right inferior frontal cortex. This observation confirms the hypothesis that face processing and adjacent areas respond automatically to the salience of faces, rather than their attractiveness or rewarding properties per se.

**Figure 3:**
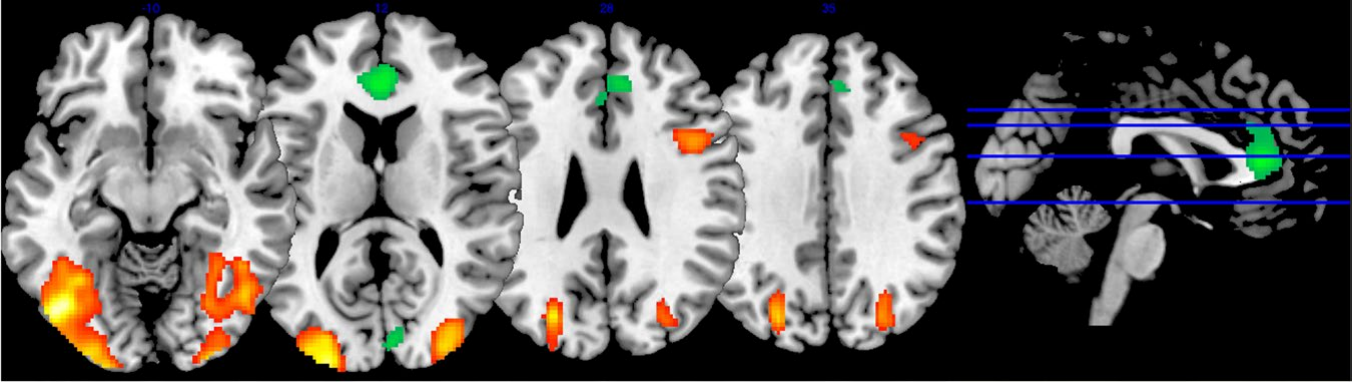
Increased activations (red yellow) and deactivations (blue-green) in response to faces before treatment. Results were corrected for multiple comparisons by familywise error correction at p < 0.05 with Monte Carlo permutation testing in SnPM with a combined cluster-voxel threshold (cluster defining threshold p < 0.001, T > 3.3852).

In addition to increased responses in visual areas, we found decreases in neural response amplitude to disfigured faces in the medial anterior cingulate gyrus extending towards medial prefrontal cortex (see Figs 2 and 3), as well as in a region stretching from right cuneus to the right calcarine gyrus and right lingual gyrus. This finding is similar to previous observations of neural responses to other stigmatized outgroups such as drug addicts and homeless people^31^ and could reflect suppression empathy and mentalizing or increased demands in cognitive control, e.g. inhibition of staring at the area of lesion or inhibition of inappropriate social behaviour like obvious avoidance. Both possible hypotheses are not mutually exclusive and could be linked to the increased activation in the left inferior frontal gyrus - a region linked to cognitive control.

In previous studies, increased amygdala activation has been reported to both positive and negative valence of faces^38,40^. Moreover, studies investigating the brain responses to extreme outgroups like homeless people and drug addicts find activations in anterior insula where it is typically interpreted as a disgust response^31^. We did not find statistically significant activations in amygdala and anterior insula. It is possible that this lack of effect is because of our smaller stimulus sample or that the difference between before and after stimulus pairs is not large enough to produce statistically significant results in this before-after contrast of the same face.

In sum, we found that people have implicit negative biases against faces that are disfigured, without being aware of harbouring such biases. Disfigured faces evoke greater neural responses in ventral occipito-temporal and right inferior frontal regions as compared to non-disfigured faces. This finding refutes the hypothesis that attractiveness and reward per se drives automatic ventral cortical responses and instead confirms the idea that ventral occipito-temporal regions are sensitive to the salience of faces.

Moreover, disfigured faces evoke lower neural responses in the anterior cingulate and medio-prefrontal cortex, as well as some visual areas. This result is similar to previously reported neural responses to stigmatized outgroups like homeless people and drug addicts^31^. In agreement with this research, we speculate that the de-activation of these brain areas upon seeing disfigured faces as opposed to the same faces after surgical treatment possibly reflects an inhibition of empathy and mentalizing or inhibition of socially inappropriate behaviour. The medial anterior cingulate gyrus and the adjacent medial prefrontal cortex are core areas of the theory of mind and empathy networks^61,62^ and are crucial for inferring other’s beliefs, feelings, and mental states. Together with previous behavioural research showing a clear association of negative personality traits and our findings of an implicit bias against disfigured faces, we take these response patterns as neural evidence for stigmatization. Future research should investigate if the de-activation of anterior cingulate cortex represents a consistent neural marker for dehumanization of people with disfigured faces or if it reflects social adaptive behaviour to people who deviate from the norm.

The emphasis of attractiveness, its association with positive attributes and robustness of these associations across cultures^5^ highlights the pervasive effect of attractiveness in social interaction. People who fall towards the lower end of the attractiveness spectrum are disadvantaged or even subject to discrimination and social isolation as in the case of facial disfigurement. Encouragingly, our findings suggest that surgical treatment of disfigurement mitigates the negative effects of disfigurement. Our findings highlight the importance of recognizing that we implicitly and automatically regard flawed faces as flawed people and that corrective surgery confers social and psychological benefits to people with facial disfigurement. Alternative prevention strategies against discrimination of disfigured people and effective support for people with facial conditions should be explored.

## Methods

### Implicit association test (IAT) and explicit bias questionnaire (EBQ)

#### Participants

80 participants were recruited via an online recruiting system for psychology experiments at the University of Pennsylvania (55 female, 25 male, mean age = 23 years, SD = 6.4, range 18–56). The sample size was determined based on estimates suggested by a meta-analysis on attitudes towards individuals with disabilities as measured by an IAT^63^. Prior to participation, participants were informed that the task was about categorising faces and words but were naïve to the fact that some of those faces might be disfigured. Participation was voluntary, and participants received money as compensation. Study procedures were approved by the Institutional Review Board (IRB) at the University of Pennsylvania (Protocol #806447). IRB approval was in accordance with the International Conference on Harmonization and the Belmont report. All participants gave written informed consent.

One participant was excluded from the data analysis for the IAT because more than 10% of the total test trials were unreasonably fast (<300 ms). After data exclusion, the data of 79 participants went into the final analysis (55 female, mean age = 23 years, SD = 6.4, range 18–56).

#### Procedure

Task order between the IAT and the EBQ was counterbalanced so that half of the participants completed the IAT first, and half of the participants completed the EBQ first. Participants were seated in a testing room, in front of a testing laptop. After having been briefed on the order of the tasks, participants gave written informed consent. The entire experiment took about 30 minutes.

The IAT^59,60^ was designed using E-Prime software and was modelled after the IATs from *Project Implicit* (https://implicit.harvard.edu). A total of 16 words were used for the IAT: 8 were positive words (attractive, happy, approachable, friendly, adore, lovely, spectacular, excellent), and 8 were negative words (ugly, evil, sickening, rotten, disaster, disgust, pain, despise).

Participants completed the EBQ as a survey on Qualtrics. Questions were modelled after the Project Implicit and Changing Faces explicit questionnaires^64^. The questionnaire included 11 questions asking about participants’ prior exposure to and conscious biases against people with facial disfigurement. Participants responded on a scale ranging from 1 to 7 (see https://osf.io/ca2u9/ for details).

#### Pictures

Images consisted of photographs of patients with facial disfigurements before and after corrective surgery. These photos were collected from craniofacial and dental surgery atlases and compilations of plastic surgery results. The disfigured faces were photos of the individuals before treatment that were affected by one of the following disfigurements: carcinoma, hyperpigmentation, birthmark, scar or small wound, facial paralysis, isolated weight loss, bone disfigurement, or facial trauma. The non-disfigured faces were photographs of the same individuals after treatment (see https://osf.io/ca2u9/ for all stimulus pairs). Pre-treatment and post-treatment photographs were cropped (to show only faces, with some hair and neck) and colour-corrected to match in size and coloring^6^. The stimulus set consisted of 28 faces, of which 22 were female and 6 were male. 16 of the faces were oriented frontally, 10 were oriented in a three-quarters portrait view, and 2 were profiles (see https://osf.io/ca2u9/).

#### Implicit association test and explicit bias measure results

Explicit scores range from −3 to +3, with zero indicating no relative preference for non-disfigured vs. disfigured faces. Positive scores indicate a preference for non-disfigured faces, and negative scores indicate a preference for disfigured faces. We found a significant implicit preference for non-disfigured faces (mean difference score = 0.90; SD = 0.58; min = −0.26; max = 2.00; *t*_(78)_ = 13.80; 95% CI = 0.77 to 1.03; p < 0.001; Cohen’s *d* = 1.55). This effect was particularly strong for male respondents (see Table 1 for details, see Fig. 1). Participants showed no significant explicit preference for non-disfigured vs. disfigured faces (mean explicit score = 0.01; SD = 0.51; min = −1.50; max = 1.08.168; *t*_(78)_ = 0.17; 95% CI = −0.11 to 0.12; p = 0.866; Cohen’s *d = *0.02). Prior exposure had no effect on bias for either the IAT or the EBQ. There was a small to moderate correlation between implicit and explicit scores that was, however, not statistically significant (Pearson’s correlation coefficient, r = 0.22; p = 0.052) making it difficult to draw conclusions as to whether people are aware of their biases.

**Table 1:** Implicit preferences for non-disfigured vs. disfigured faces for all participants by gender.

	N (%)	Mean Implicit Preference (a)	SD	t	p	Min	Max	Cohen’s *d* (b)
**All Participants**	79	0.90	0.58	13.80	<0.001	−0.26	2.00	1.55
**By Gender**								
Male	24 (30.4)	1.18	0.52	11.25	<0.001	0.07	2.00	2.30
Female	55 (69.6)	0.77	0.56	10.18	<0.001	−0.26	2.00	1.37
**Paired samples** ***t*** **-test**		**Mean difference**		**t**	**p**			**Cohen’s** ***d***
		0.55		3.47	0.002			0.71

^a^IAT D scores range from −2 to +2, with zero indicating no relative preference for non-disfigured vs. disfigured faces. Positive scores indicate an implicit preference for non-disfigured faces while negative scores indicate an implicit preference for disfigured faces. D scores were interpreted according to specific, conservative break points based on Cohen’s *d*: ±0.15 (‘slight’ bias), 0.35 (‘moderate’ bias), 0.65 (‘strong’ bias).

^b^Cohen’s *d* is a standardized effect size, interpreted as *d* of 0.2 = small effect, *d* of 0.5 = medium effect, and *d* ≥ 0.8 = large effect.

### FMRI experiment

#### Participants

We recruited 34 healthy right-handed college students from University of Pennsylvania (24 females, 10 males). Age of participants ranged from 18–35 years. Participants had normal or corrected to normal vision and no prior history of psychiatric or neurological disease. Before participation in the study, each individual gave informed consent approved by the IRB at the University of Pennsylvania (Protocol #806447) in accordance with the International Conference on Harmonization and the Belmont report.

The data of three participants was excluded from the final analysis. One dataset was excluded because of technical failure which stopped the stimulus presentation halfway through the experiment. Two other datasets were excluded because of synchronization problems between experimenter laptop and the scanner triggers. The data of 31 participants entered the final analysis (22 females, 9 males).

The EBQ for the participants in the fMRI experiment showed that about half of the participants have a close friend or family member with either a facial disfigurement or a disability. Exposure to people with facial disfigurement was normally distributed in the sample, and most participants reported no to slight preference for non-disfigured over disfigured people (22/28 data entries, 5 missing values).

#### Procedure and stimulus presentation

The experiment consisted of one session. Participants were presented with 28 pictures of faces in randomized order and were asked to decide whether the displayed face was male or female. Half of the presented pictures were photographs of patients before treatment, and half after treatment. The pictures were identical to the ones used in the behavioural experiment (IAT, see above). Stimuli were presented using E-prime software by projecting them onto a screen using a projector outside the MR scanner room, which could be seen by participants through a mirror mounted over the head coil. Each picture was presented for 6 seconds. Responses were recorded with a 2-button response device. After the experiment, a high-resolution anatomical scan (~7 min) was conducted. After the scanning session, participants were taken out of the scanner and completed the EBQ for disfigurement on a testing computer outside the scanner room. This test was identical to the EBQ in the online sample reported above.

#### fMRI data acquisition and pre-processing

Images of blood-oxygen level dependent (BOLD) changes were acquired with a 3 T Siemens Magnetom Prisma scanner (Erlangen, Germany) with a 64-channel head coil. We used cushions to minimize participants’ head movement. We used two localizing scans and auto-alignment. Functional images were acquired using a standard BOLD sequence (TR: 2000 ms, TE: 30 ms, flip angle: 60 degrees, voxel size: 2.0 × 2.0 × 2.0 mm, 81 slices). High resolution (0.8 × 0.8 × 0.8 mm) structural (anatomical) images were acquired using an SPC T1 GRAPPA sequence^65^. Data were pre-processed using the Matlab toolbox SPM12 (http://www.fil.ion.ucl.ac.uk/spm). Images were motion corrected and registered to the first image of the scanning block. The mean of the motion-corrected images was co-registered with the individual participants’ anatomical scan. The anatomical and functional scans were spatially normalized to the standard MNI template. Finally, all data were spatially smoothed using an isotropic 8 mm full width at half maximum (FWHM) Gaussian kernel.

#### fMRI data analysis

At the single-subject level, statistical analysis was performed using a general linear model. The motion estimates of the motion correction algorithm were modelled as regressors of no interest to account for head motion. We performed a whole-brain group analysis by directly contrasting the mean activations per condition in a non-parametric design with SnPM (https://warwick.ac.uk/fac/sci/statistics/staff/academic-research/nichols/software/snpm). Results were corrected for multiple comparisons with a combined voxel-cluster level threshold by familywise error correction at p < 0.05 with Monte Carlo permutation testing.

In addition to the whole brain group analysis, we performed an item-wise region of interest control analysis to test if the effects in the group analysis are driven by specific items. The two clusters were defined by the group contrasts in the whole brain analysis and consisted of one area comprising of the two large occipital activation clusters, and one comprising the (de-)activation cluster in the anterior cingulate cortex. Mean values for these two regions were extracted for each subject and item. The mean values were normalised with the individual subject’s mean activation in this area to create relative difference scores per subject and item. The data for the item-wise analysis were analysed with linear mixed effect models in RStudio. We built one base model for each dependent variable (occipital cluster activation, anterior cingulate cluster activation) that included condition (pre or post surgery picture) as a predictor and subject and item as random factors with random intercepts. We tested for both random factors whether including random slopes for the condition would improve the model fit and tested interactions with gender and EBQ responses with the best base model (see https://osf.io/ca2u9/ for details).

#### FMRI sample results

Participants performed at ceiling for the gender judgment task.

**Figure 4:**
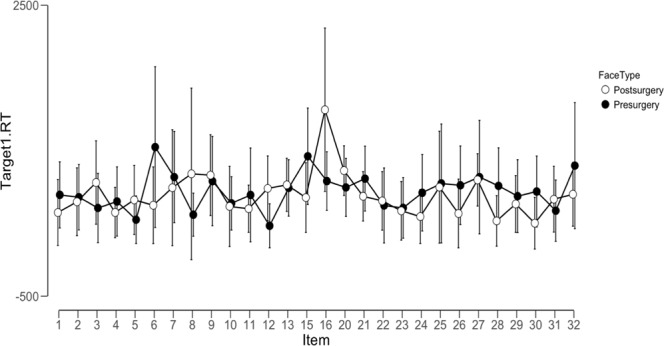
Reaction times for gender judgement task per item split by face type. Error bars display 95% confidence intervals.

An ANOVA analysis of the reaction time data in the gender judgement task in the scanner revealed no differences in reaction times between before and after treatment pictures (F_(1)_ = 0.56, p = 0.45, see Fig. 4) and no differences for item (F_(27)_ = 1.26, p = 0.17) and no interaction between item and face type (F_(27)_ = 1.06, p = 0.38).

We found increased activations in temporo-occipital regions encompassing bilateral middle occipital and fusiform gyrus, left inferior occipital gyrus, as well as right inferior temporal and right inferior frontal gyrus (Fig. 2; see Table 2 for details). An area in the medial anterior cingulate cortex and an area in the right calcarine gyrus showed significant decrease in activation in response to faces before surgery (Fig. 2; see Table 3 for details). All clusters statistically significant at p < 0.05 FWE at the cluster level corrected by Monte Carlo permutation testing (cluster forming threshold p < 0.001 per voxel).

**Table 2:** Increased responses to faces before treatment, familywise error corrected with Monte Carlo permutation testing.

Location	k	T-max	x	y	z
left lateral occipital gyrus/BA 18	3442	8.08	−28	−98	8
right lateral occipital gyrus/BA 18	2377	6.98	34	−90	2
right inferior frontal gyrus/BA 44	230	5.02	42	8	26

**Table 3:** Decreased responses to faces before treatment, familywise error corrected with Monte Carlo permutation testing.

Location	k	T-max	x	y	z
left and right anterior cingulate cortex/BA 24	765	4.92	−2	36	10
right calcarine gyrus/BA 18	247	4.10	6	−88	12

**Figure 5:**
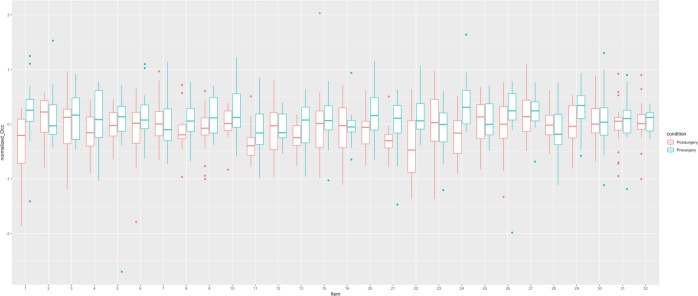
Itemwise mean activation in the occipital cortex. Stimulus items that do not follow the general activation pattern are Item 2, 7, 12, 25, and 28.

**Figure 6:**
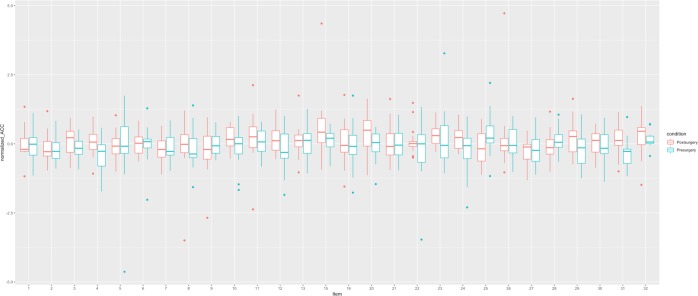
Itemwise mean activation in the anterior cingulate cortex. Stimulus items that do not follow the general activation pattern are Item 1, 25, and 28.

The ROI analysis controlling for random effects of items and subjects confirmed the results of the whole brain analysis (see Figs 5 and 6, see https://osf.io/ca2u9/ for analysis code and full statistical details). Whether the picture of a person was presented from before or after surgery had a significant effect on the BOLD activation level in the anterior cingulate cluster (β = −0.15, s.e. = 0.05, t = −2.95), as well as the occipital cortex (β = 0.17, s.e. = 0.03, t = 5.31). Neither gender of the participant, any of the EBQ measures (see Tables 4 and 5 for descriptive statistics), or the gender of the depicted person was found to be related to BOLD activation level differences.

**Table 4:** Summary of the EBQ responses I.

	Exposure	Warm vs cold	Preference	They are more happy, confident, assured, and cheerful than others.	They are more sad, shy, and miserable than others.	They are more attractive, desirable, and eligible than others.	They are more unattractive, undesirable, and unsuitable than others.	They are more easy-going, approachable, likeable, and friendly than others.	They are more awkward, unlikeable, unapproachable, and unfriendly than others.	They are more successful, motivated, accomplished, and more likely to succeed than others.	They are more limited and unmotivated and more likely to fail than others.
**Descriptive Statistics**											
Valid	28	28	28	27	27	25	26	27	25	28	27
Missing	5	5	5	6	6	8	7	6	8	5	6
Mean	2.714	4.464	−0.7143	3.185	4.259	3.040	3.615	3.963	3.040	3.750	2.815
Std. D.	0.8968	0.9616	0.9759	0.9214	1.130	1.098	1.551	0.8979	1.207	1.041	1.360
Min.	1.000	3.000	−3.000	1.000	1.000	1.000	1.000	2.000	1.000	1.000	1.000
Max.	4.000	7.000	0.000	4.000	6.000	5.000	6.000	6.000	5.000	5.000	5.000

**Table 5:** Summary of the EBQ responses II.

	Sad (1) - Hap-py (7)	Unconfi-dent (1) - Confide-nt (7)	Incomp-etent (1) - Competent (7)	Shy (1) - Assu-red (7)	Miser-able (1) - Chee-rful (7)	Unattra-ctive (1) - Attracti-ve (7)	Undesir-able (1) - Desirab-le (7)	Ugly (1) - Gorge-ous (7)	Stupid (1) - Intelli-gent (7)	Unsuit-able (1) - Eligible (7)	Awkward (1) - Easy-going (7)	Untrustw-orthy (1) - Trustwor-thy (7)	Unapproa-chable (1) - Approach-able (7)	Unfrie-ndly (1) - Friendly (7)	Non-achiever (1) - Achiever (7)	Ordinary (1) - Accompl-ished (7)	Unmoti-vated (1) - Motivat-ed (7)
**Descriptive Statistics**																	
Valid	28	27	27	27	28	28	27	28	28	28	28	28	28	28	28	28	28
Missing	5	6	6	6	5	5	6	5	5	5	5	5	5	5	5	5	5
Mean	3.786	3.444	4.926	3.704	3.929	3.464	3.667	3.679	4.571	4.393	4.143	4.571	3.929	4.393	4.464	4.143	4.429
Std. D.	0.8325	0.8006	1.141	0.6086	0.6042	0.9993	0.8321	0.7724	1.034	1.166	0.9705	0.9974	1.052	1.031	0.9993	0.8483	0.9595
Min.	2.000	2.000	4.000	3.000	3.000	2.000	2.000	2.000	4.000	3.000	2.000	4.000	1.000	2.000	3.000	1.000	3.000
Max.	6.000	5.000	7.000	5.000	5.000	7.000	6.000	5.000	7.000	7.000	6.000	7.000	6.000	7.000	7.000	6.000	7.000

## Data Availability

The datasets generated and analysed during the current study will be made available without restriction on Open Science Framework (DOI 10.17605/OSF.IO/CA2U9) upon acceptance of the article for publication, https://osf.io/ca2u9/.
